# Study on Performance Tests and the Application of Construction Waste as Subgrade Backfill

**DOI:** 10.3390/ma14092381

**Published:** 2021-05-03

**Authors:** Qingbiao Wang, Jie Zhang, Kang Liu, Andong Xu, Haolin Xu, Mingcong Yang, Cun Wang, Rongshuai Yang, Guangtao Bao, Yunfei Liu, Zhongjing Hu, Zhenyue Shi

**Affiliations:** 1 State Key Laboratory of Mining Disaster Prevention and Control Co-Founded by Shandong Province and the Ministry of Science and Technology, Shandong University of Science and Technology, Qingdao 266590, China; skd990748@sdust.edu.cn; 2 National Engineering Laboratory for Coalmine Backfilling Mining, Shandong University of Science and Technology, Tai’an 271019, China; 3 Department of Resources and Civil Engineering, Shandong University of Science and Technology, Tai’an 271019, China; 4 College of Civil Engineering and Architecture, Shandong University of Science and Technology, Qingdao 266590, China; 201882040019@sdust.edu.cn; 5 College of Resources, Shandong University of Science and Technology, Tai’an 271019, China; 202083300011@sdust.edu.cn (K.L.); 202083300023@sdust.edu.cn (A.X.); 202083300024@sdust.edu.cn (H.X.); 202083300025@sdust.edu.cn (M.Y.); 202083300036@sdust.edu.cn (C.W.); 202083300026@sdust.edu.cn (R.Y.); 202083300002@sdust.edu.cn (G.B.); 202083300013@sdust.edu.cn (Y.L.); 6 College of Safety and Environmental Engineering (College of Safety and Emergency Managemen), Shandong University of Science and Technology, Qingdao 266590, China; huyang@sdust.edu.cn

**Keywords:** LFCWM, compaction test, CBR value, shear strength

## Abstract

The application of construction waste as an aggregate in subgrade backfilling is an important recycling option. This study analyzed a subgrade backfill material consisting of lime-fly ash construction waste mixture (LFCWM). Compaction and California bearing ratio (CBR) tests were performed on LFCWM under different cement-aggregate ratios (CARs, 3:7, 4:6, 5:5, 8:2). Different normal stresses (100, 200, and 300 kPa) and aggregate sizes (20%, 40%, 60%, 80% of P_4.75_) were also evaluated. The experimental results indicated that: (1) when the CAR was 4:6, the optimum water content and the maximum dry density reached their maximum values of 10.1% and 2.03 g/cm^3^, respectively, the maximum CBR value was 42.5%, and the shear strength reached its maximum value. (2) With an increase in shear displacement, the shear stress showed a rapid initial increase, then a slow decrease, and finally tended to stabilize. (3) Normal stress had a positive effect on the shear strength of the mixture. (4) When P_4.75_ was 40%, the shear strength of LFCWM was the maximum. The research results have been successfully applied to road engineering, providing an important reference for the application of construction waste aggregate in roadbed engineering.

## 1. Introduction

Construction waste is a waste resource with a wide range of applications [1]. Using construction waste as a subgrade backfill is a more direct method of application, which can effectively reduce the consumption of natural resources such as gravel. Therefore, it is necessary to actively explore methods and promote the application of construction waste in subgrade engineering.

Wang et al. and Wu et al. [2,3] applied construction waste in recycled brick, Yu et al. and Luan et al. [4,5] applied it in sheet metal, Ju et al., Chen et al., Si et al., and Wu et al. [6,7,8,9,10] replaced traditional aggregate in concrete by construction waste, and Wang et al. and Yang et al. [11,12,13] studied its application as subgrade filling. Construction waste is widely used in many fields, and it is also used in subgrade backfill.

Considering studies on subgrade filling, Wang et al. [14] studied the application of a natural cinder and fine granular soil mixture in subgrade in China. Luo et al. [15] studied the consolidated undrained shear strength, failure potential, and CBR of a cinder gravel and silt mixture. Zeng et al. [16] studied the working principle of nanomaterials and their advantages in roadbed and pavement engineering, Liu et al. [17] discussed the possibility of improving high plastic soil with crushed rock and studied the effect of crushed rock grade on the elastic modulus of a soil crushed rock mixture. In addition, Zhang et al. [18] analyzed the physical and chemical properties of CDW (construction and demolition waste) materials for expressways, studied the construction technology of the CDW subgrade, and performed a series of tests. Zhang et al. [19] revealed the permanent deformation response of subgrade construction and demolition materials under different water content, compaction, deviator stress, and confining pressure through a dynamic triaxial test. Chen et al. [20] evaluated the feasibility of using cement waste as a filling material for loess roadbeds. The research on subgrade backfill materials is relatively rich, involving gravel, coal cinder, cement, soil, and other materials, among which gravel, cement, and soil are the most commonly used backfill materials.

Regarding the application of construction waste aggregates in roadbed engineering, Boye et al. [21] studied the performance of construction waste on flexible and rigid road surfaces and the impact of recycling such waste. Debnath et al. [22] studied the blocking performance of pervious concrete made of waste and established a prediction model to understand the blocking characteristics of pervious concrete pavement. Vieira et al. [23] investigated the feasibility of using fine-grained construction and demolition recycled materials as backfill for geosynthetic-reinforced earth structures (embankments and retaining walls) to replace the soil normally used in these structures. Liang et al. [24] studied the use of construction waste clay brick instead of cement to stabilize the fine aggregate of a graded gravel base at a specific proportion.

In summary, construction waste aggregates have a wide range of applications and have been applied in subgrade backfill engineering. At present, commonly used roadbed backfill materials are mainly crushed stone, cement, and soil. Studies have also covered cinder mixed with soil and construction waste mixed with soil. However, few studies focused on the application of construction waste, fly ash, and other waste materials. Materials such as fly ash, lime, and construction waste are more convenient as they are easy to acquire. In addition, lime has a simple production and is a low-cost material, thus its use replacing cement can provide large cost savings. Fly ash is an easily available industrial waste, and is used replacing soil and silt, which can prevent the destruction and waste of natural resources.

At present, research on the use of fly ash, lime, and construction waste aggregate as subgrade backfill materials has not been performed. Based on the engineering requirements, in this study, laboratory tests such as the compaction test, CBR test, and shear test of LFCWM were conducted. In addition, the effects of the CAR on the dry density, moisture content, CBR, and shear strength were analyzed. The effects of normal stress and particle size of construction waste on the shear strength were also studied. The research results have been applied in road engineering of the Jinan East Railway Station Comprehensive Transportation Hub. The bearing capacity obtained in the test was in agreement with the design requirements, and the application effect was good, which provides an important reference for the application of construction waste mixtures in roadbed engineering.

## 2. Performance Test of LFCWM

### 2.1. Preparation of Experimental Materials

**Figure 1:**
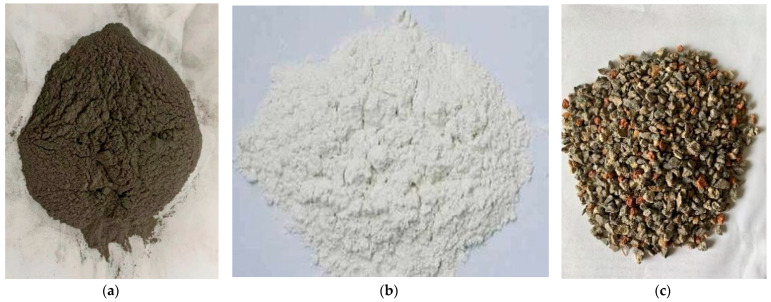
(**a**) fly ash; (**b**)lime; (**c**) construction waste aggregate.

Secondary fly ash was produced by Gongyi Bolun Refractory Co., Ltd (Gongyi, China). The characteristic parameters are listed in Table 1. Lime was produced by the Maoshun Lime Factory, Jioxian County, Dongping County, Tai’an. Its characteristic parameters are listed in Table 2. The construction waste aggregate used in this test was mainly composed of concrete blocks, bricks, and stones, with particle sizes between 2 and 5 mm. Samples of these materials are shown in Figure 1.

### 2.2. Test Sample Preparation and Experimental Method

In this test, the mixture was composed of fly ash, lime, and construction waste, and the mixing ratio of fly ash and lime was 1:2. Therefore, a sufficient weight of fly ash and lime mixture was configured in this proportion to be mixed with the construction waste. In addition, the CAR refers to the mass ratio of lime and fly ash mixture to construction waste aggregate.

#### 2.2.1. Compaction Test Specimen Preparation and Experimental Method

(1) Compaction test specimen preparation

**Figure 2:**
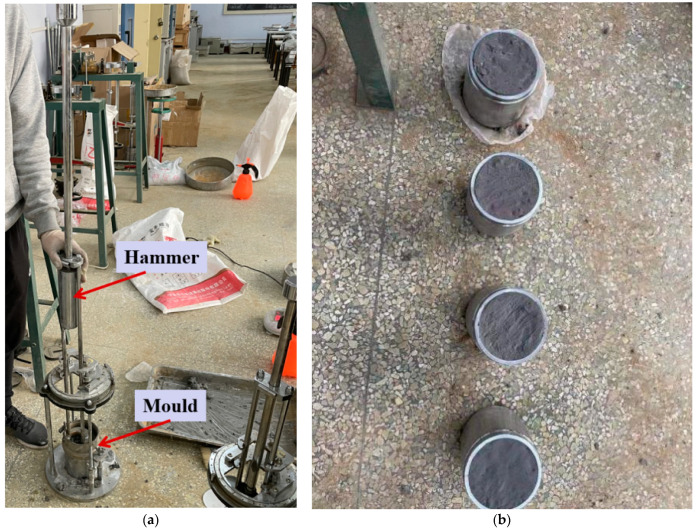
Compaction experiment: (**a**) compaction test equipment; (**b**) compaction test sample preparation.

In the compaction test, four subgrade backfill materials with CARs of 3:7, 4:6, 5:5, and 8:2 were tested. Each CAR condition contained five water content experimental groups, with water contents of 6%, 8%, 10%, 12%, and 14%. The design of the experimental group is shown in Table 3, where “A” indicates that there was one sample in the test group. The compaction test samples were prepared using a JDS-3 standard portable compaction instrument. The instrument mold was a cylinder with an inner diameter of 102 mm and a height of 116 mm, as shown in Figure 2. The configured construction waste mixture was poured into the mold in three layers. After pouring a layer of the mixture, a 2.5 kg hammer was used to compact the mixture in the mold. The hammer was lift 30 cm and released to compact the mixture, and this was performed 27 times [25]. This process was repeated for each layer. After preparing the sample, the moisture content test was performed.

(2) Compaction test method

The sample was removed from the cylinder with a bulldozer, and the central part of the sample was taken as the representative sample for the water content test. The dry density was calculated using the formula ρd = ρ/(1 + 0.01ω), where ρd is the dry density; ρ is the wet density, which can be calculated by the relationship between mass and volume; and ω is the moisture content.

#### 2.2.2. Preparation and Experimental Method of CBR Test Specimen

(1) CBR test sample preparation

**Figure 3:**
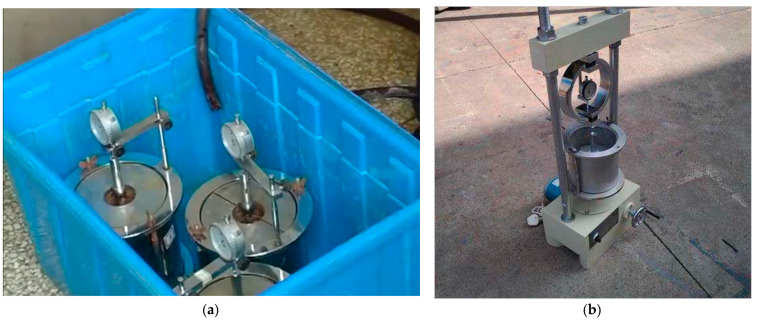
Load-bearing ratio test: (**a**) soak sample; (**b**) bearing ratio test equipment.

Through the compaction test, we obtained the optimal water content corresponding to CARs of 3:7, 4:6, 5:5, and 8:2 as 10%, 12%, 12%, and 12%, respectively. The setting of CBR experimental group is shown in Table 4, where A, B, and C indicate the samples in each group. CBR samples were also prepared with a JDS-3 standard portable compaction instrument, but the instrument mold was selected as a 152 mm inner diameter cylinder with a height of 170 mm [25]. After producing the mixture according to the ratio shown in Table 4, the samples were produced. The production method was the same as that used in the compaction test. As is shown in the Figure 3a Compacted samples, plus the load plate, were placed in a water tank to soak for four days and nights to fully absorb water, after which the CBR test was performed.

(2) Test method for CBR

The CBR test adopted a BR-1 bearing ratio tester, as shown in Figure 3b. The soaked sample was removed from the tank and placed on the instrument. The penetration rod was pressed into the sample at a speed of 1.00–1.25 mm/min, recording the penetration amount using the internal dial gauge. When the penetration amount was 2.5 mm, more than five readings were taken. The ratio of the pressure and the standard pressure (standard pressure = 7000 kPa) when the penetration volume is 2.5 mm is used as the CBR, and the final CBR value is the average value.

#### 2.2.3. Preparation of Shear Test Samples and Experimental Methods

(1) Preparation of shear test samples

The fly ash, lime, and construction waste aggregate were mixed according to the CARs shown in Table 5. The earth collector was used to extract part of the mixture from the compacted mixture, and then the ring knife was used to insert the mixture into a mold with a diameter of 61.8 mm and a height of 20 mm [25].

According to the compaction test of the construction waste backfill, when the CAR of the mixture was 4:6, the optimum moisture content was the lowest, and the dry density was the highest. Therefore, the effect of normal stress on shear strength was studied for the CAR of 4:6. The test of the influence of normal stress on shear strength was performed according to the scheme shown in Table 6, and the soil collector and ring cutter were used for sampling.

The influence of the particle diameter on the shear strength was tested in accordance with the scheme shown in Table 7. The diameter of 4.75 mm is the boundary to distinguish coarse and fine particles. P4_.75_ represents the proportion of aggregates with particle sizes higher than 4.75 mm in the aggregate used. The recommended range of P_4.75_ for stably graded gravel of fly ash is 30–50%, Thus, P_4.75_ was set to 20%, 40%, 60%, and 80% [26]. After the material was proportioned and mixed, the samples were taken with a soil collector and a ring cutter.

(2) Shear test method

**Figure 4:**
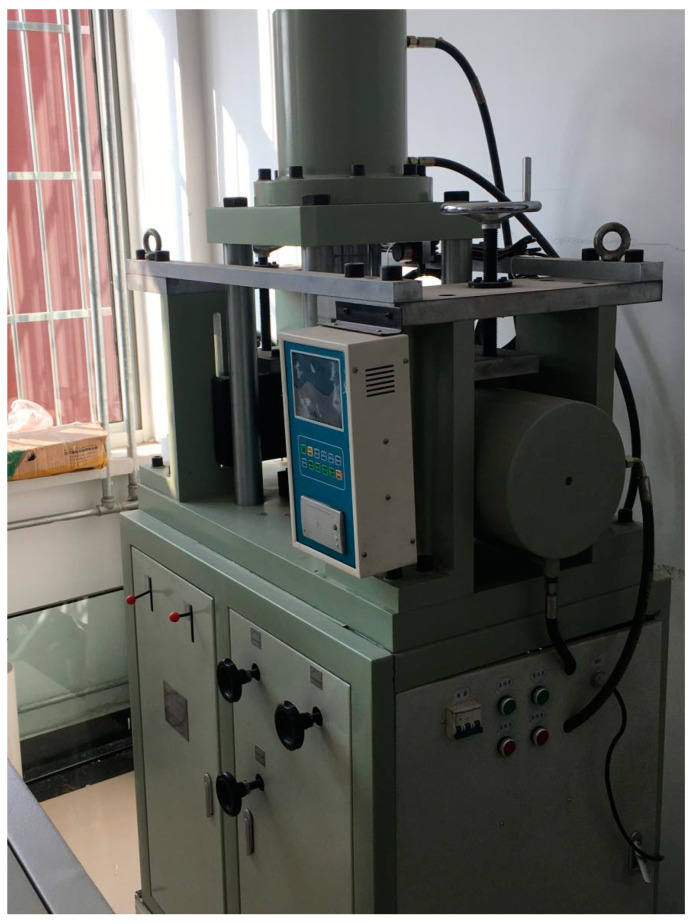
Direct shear instrument.

The sampled ring knife was placed on the shear box, which was placed on the direct shear instrument, as shown in Figure 4. The load value in the generator was used to control the pressure requirement. The shear rate was controlled at 0.5 mm/min. During the shearing process, data such as shear stress and shear displacement were collected through a built-in 4-channel data collector.

## 3. Results and Discussion

The experimental results of compaction, CBR, and shear strength of each test group were obtained using the test methods described, and the results, consisting of mean values of the three specimens in each group, were displayed in graphs and systematically analyzed.

### 3.1. Compaction Test Results Analysis

**Figure 5:**
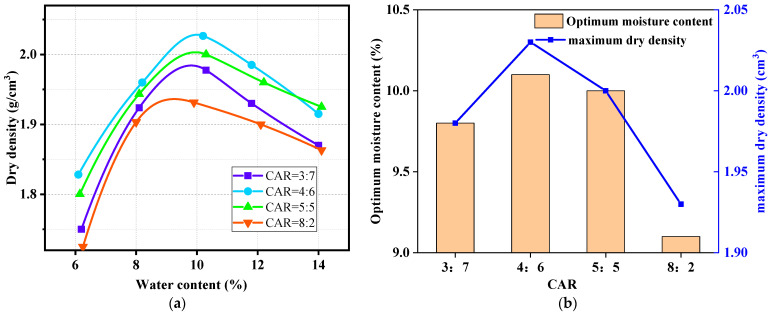
(**a**) Relationship curve between moisture content and dry density; (**b**) optimum moisture content and maximum dry density corresponding to different CARs.

Through the compaction test, the relationship between water content and dry density of LFCWM under different CARs was obtained, as shown in Figure 5.

As shown in Figure 5a, the dry density increased first and then decreased with the increase in water content, and the increasing tendency was relatively fast, whereas the decreasing tendency was slow. The moisture content and dry density lines were in accordance with the standard reference from [25]. When the CARs were 3:7, 4:6, 5:5, and 8:2, the optimal water content and maximum dry density were (9.8, 1.98), (10.1, 2.03), (10, 2), and (9.1, 1.93), respectively. The relationship between the optimal moisture content and the maximum dry density under the four CARs is shown in Figure 5b. According to Figure 5b, with an increase in CAR, both the optimal moisture content and maximum dry density first increased and then decreased; when the CAR was 4:6, both the optimal moisture content and the maximum dry density reached their maximum simultaneously. Zhou et al. obtained a similar result for silt in the compaction test [27].

The reasons leading to the change tendency of the optimal moisture content are as follows: when the compaction test is performed for a small the water content of the mixture, the movement range of the particles will be small, leading to a smaller compactness, and then the dry density is reduced; with an increase in water content, the thickness of the bound water film increases, the friction between particles decreases, the dislocation range is larger, the compactness is larger, and the dry density is larger. If the water content reaches the critical point, the LFCWM becomes saturated, and water will squeeze out the air entrapped. The pores entrapped by the water film in the particles are in a closed state, and water and air are sealed inside the void. Then, the bound water film between particles will bear part of the compaction work, reducing the active work between particles and the maximum dry density.

The reasons for the change in the maximum dry density are as follows: when the amount of construction waste aggregate increases, the contact between the coarse particles gradually assumes a skeleton function [28,29], and the lime-fly ash (LFA) fills in the middle of the pores. When the two reach a certain proportion limit, the optimal dry density of the mixture reaches a maximum. With a further increase in the mixing amount of construction waste aggregates, LFA is not sufficient to fill the pores between construction waste aggregates, resulting in the formation of overhead space between construction waste aggregates and a decrease in the optimal dry density of the mixture.

### 3.2. Analysis of Bearing Ratio Test Results

**Figure 6:**
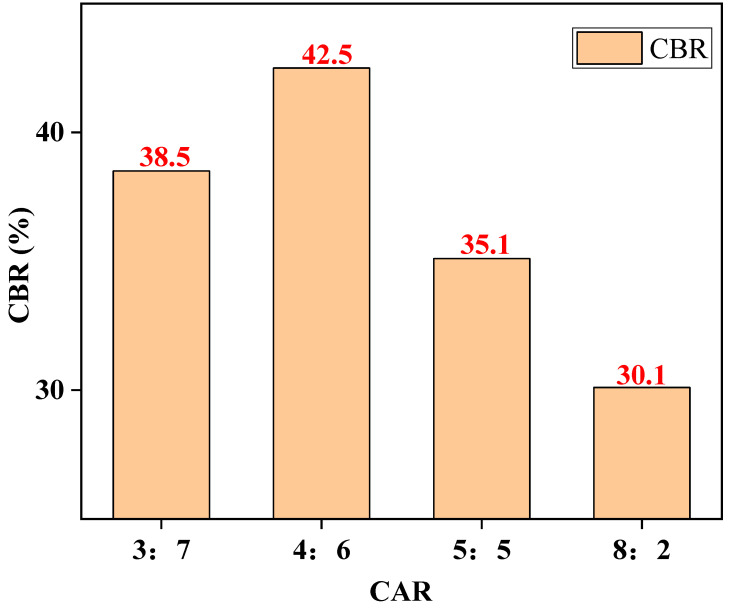
CBR values for different CARs.

As can be seen from Figure 6, when the CARs were 3:7, 4:6, 5:5, and 8:2, the CBR value of LFCWM showed a tendency of first increasing and then decreasing with the increase in construction waste aggregate incorporation. When the construction waste aggregate incorporation reached 60%, the CBR value reached a maximum. After compaction of the mixture, the particles are recombined, and there are changes in the tightness between the particles, pores, unit mass of the mixture, and overall strength of the mixture, resulting in a corresponding change in the CBR. With an increase in the waste aggregate content, the coarse aggregate content in the mixture gradually increased, the mixtures filled each other, and the gradation ratio gradually reached the optimal value. After the critical value, the CBR decreased with the deterioration of the gradation ratio.

### 3.3. Analysis of Shear Strength Test Results of LFCWM

#### 3.3.1. Influence Analysis of Shear Strength of LFCWM with Different CARs

**Figure 7:**
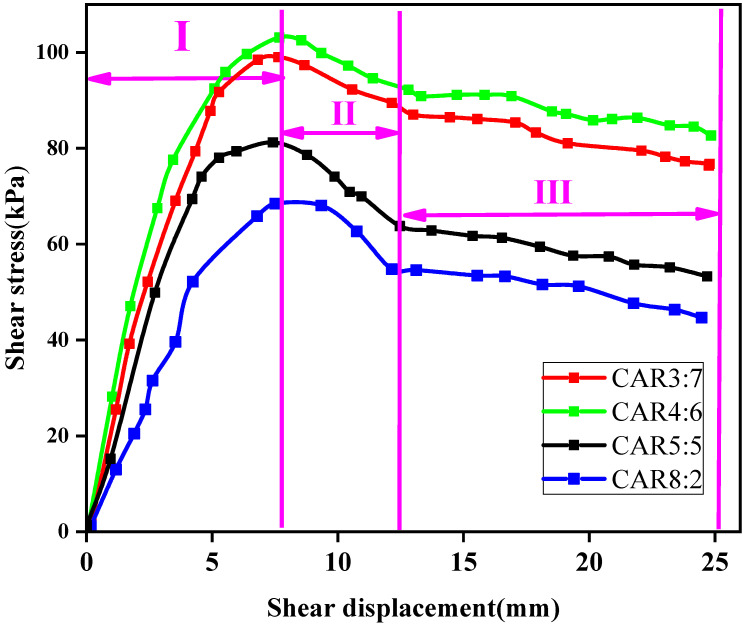
Shear stress-displacement curves for different CARs when the normal stress is 100 kPa.

It can be seen from Figure 7 that under the condition of a fixed normal stress of 100 kPa, when the CARs were 3:7, 4:6, 5:5, and 8:2, the maximum shear strengths of the mixtures were 99.02, 103.13, 81.21, and 68.45 kPa, respectively. With the increase in shear displacement, the variation tendency of shear stress increased rapidly at first, then decreased slowly, and finally tended to be stable (or with a small variation). Therefore, the shear stress-displacement curve can be divided into three stages. The first stage is the energy storage stage, in which the shear stress-displacement changes approximately linearly. The gap of the mixture gradually becomes compact due to compression, and the mixture has not been damaged; therefore, the shear displacement is small, and the shear stress increases rapidly. The second stage is the failure stage. After the shear strengthening of the mixture, shear failure occurs. Owing to the large roughness of the contact surface formed by the initial failure, the shear stress decreases slowly during the process of relative displacement. The third stage is the stable friction stage. When the upper and lower failure surfaces run in to each other and reach a stable state, the friction coefficient is relatively fixed, the shear stress gradually tends to be stable, and the curve shows a relatively stable or slightly changing tendency. Construction waste aggregates had a positive effect in the shear resistance process, which prevented shear damage to the contact surface. After damage, the contact friction can be increased, and the shear strength of the material can be improved.

At the same time, the maximum shear strength increased first and then decreased with the increase in the CAR, and reached a maximum when the CAR was 4:6. This is because after adding construction waste aggregate, the construction waste aggregate inside the mixture forms a mosaic structure, which increases the shear strength [30]. However, with the increase in construction waste aggregate, the structure changes from a mosaic structure to a contact structure of the aggregate, which forms a weak surface and reduces the shear strength.

#### 3.3.2. Influence Analysis of Different Normal Stresses on Shear Strength of LFCWM

**Figure 8:**
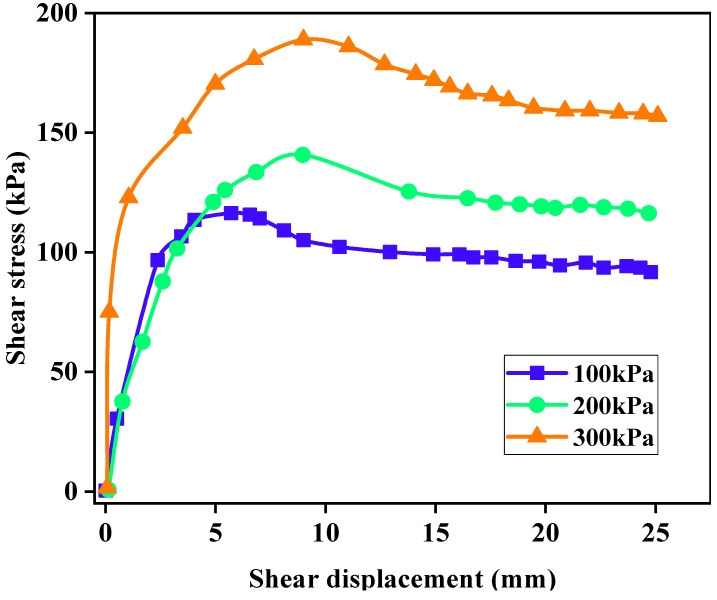
Shear stress-displacement curves of different normal stresses when the CAR was 4:6.

According to Figure 8, when the CAR of the mixture was 4:6, the maximum shear strength of the mixture when the normal stresses were 100, 200, and 300 kPa were 116.29, 140.27, and 188.80 kPa, respectively. The shear strength of the mixture increased with an increase in normal stress. This is because when the normal stress increases, the LFA particles and construction waste aggregate particles squeeze each other, which increases the cohesion force and thus increases the shear strength. When the shear strength reaches the maximum and enters the stable friction stage, the normal stress increases the contact pressure of the upper and lower failure surfaces, such that the shear stress also increases with an increase in the normal stress.

#### 3.3.3. Influence of Particle Size on Shear Strength of LFCWM

**Figure 9:**
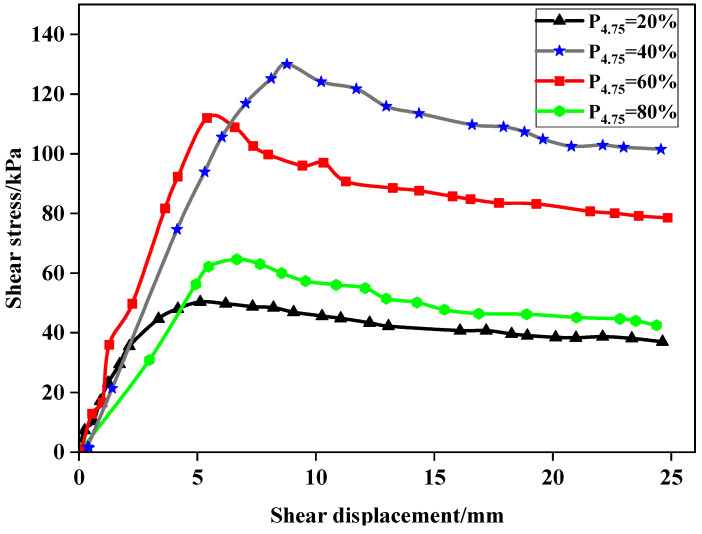
Shear stress-displacement curves of construction waste aggregates with different particle sizes at a normal stress of 100 kPa.

The influence of particle size on the shear strength of LFCWM was studied under a shear stress of 100 kPa. As shown in Figure 9, with the increase in shear displacement, the variation tendency of shear stress increased rapidly at first, then decreased slowly, and finally tended to be stable. With an increase in the amount of coarse aggregate, the shear strength of the mixture increased and then decreased. When P_4.75_ was 20%, 40%, 60%, and 80%, the corresponding maximum shear strengths of the mixed material were 50.29, 129.97, 111.93, and 64.58 kPa, respectively. It can be seen that when P_4.75_ was 40%, the shear strength of LFCWM was the largest. This is because the gradation of LFCWM changes owing to the change in the particle size of the recycled aggregate. When the aggregate particle size is small, the gradation effect is weak; thus, the strength of the aggregate itself has not a full effect. When the aggregate size is too large, the mosaic effect changes to a contact effect, and the contact structure forms a weak surface, which reduces the shear strength.

## 4. Engineering Application

**Figure 10:**
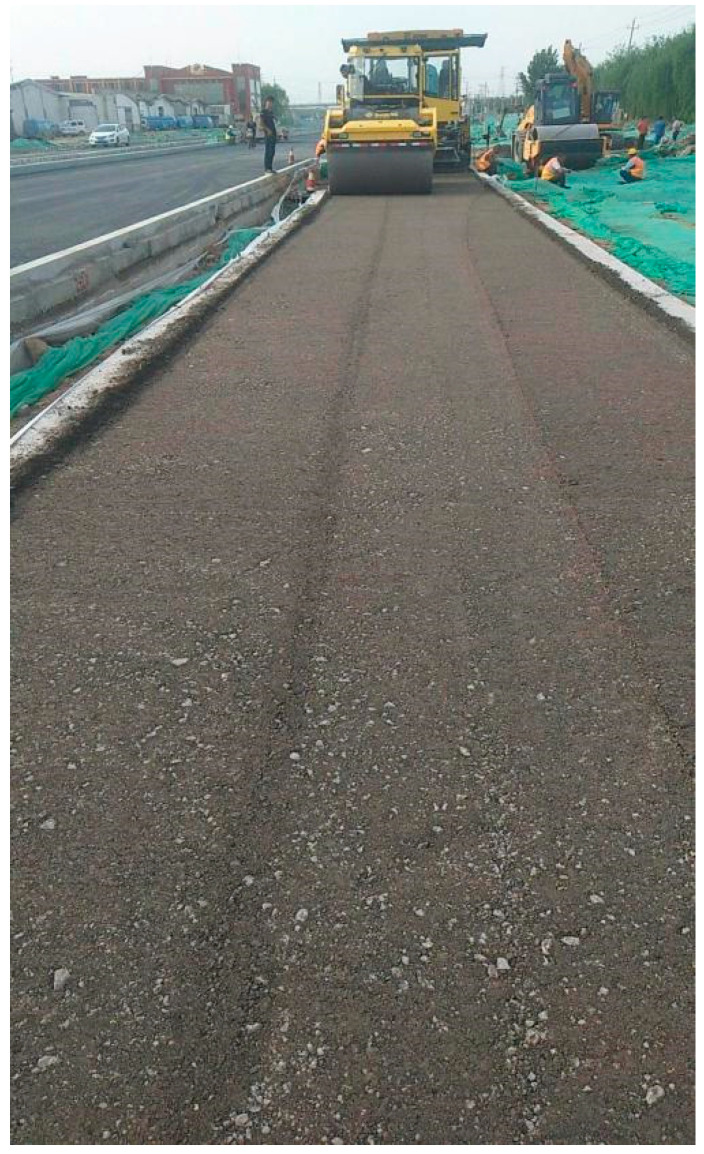
Photos of construction site.

Jinan East Railway Station Integrated Communication Hub Inward and Outward Road Project Part of the road project is located on the west side of the existing Kaiyuan Road, starting from Kaiyuan Road in the south and ending at the west approach section of Jinan West Railway Station in the north. The starting and ending pile numbers were K0+000 and K3+316.209, respectively. The road length is 3316.2 m and the width is 35 m, located on the west side of the existing Kaiyuan Road. As is shown in the Figure 10.

In an urban road roadbed, by adopting construction waste filling aggregate, the cost and construction waste caused by the occupation of land can be reduced, as well as problems such as water pollution and air pollution, in line with green development.

### 4.1. Construction Scheme

Because the mixture contains construction waste filling aggregate, the traditional construction technology may lead to insufficient rolling strength and high moisture content of construction waste aggregate, resulting in roadbed instability, service life reduction, and other problems. Therefore, construction should be avoided during the rainy season. If the construction is performed during the rainy season, the roadbed should be well protected against drainage. In the spreading process, the size of the grid was 8 m × 8 m, the virtual spreading coefficient was controlled at 1.18, the virtual spreading thickness was 350 mm, and the rolling method was vibration rolling.

### 4.2. Test Results and Analysis of Deflection Value

The bending settlement value refers to the total vertical deformation or vertical rebound deformation value generated by the wheel clearance position on the subgrade surface under a certain vertical load under relevant standards, with 0.01 mm as the basic unit. The completion acceptance deflection value is mainly used to measure whether the roadbed meets the design standards [27]. The carrying capacity of the material was characterized. To obtain more accurate data, a drop weight bending and sinking instrument was used in this test, and the test results were compared and analyzed.

**Figure 11:**
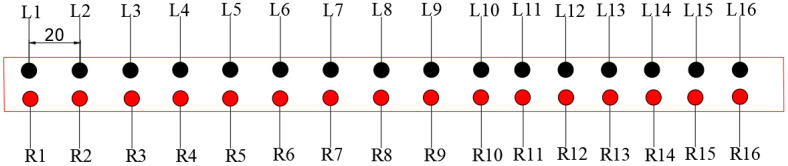
Arrangement of monitoring points for bending settlement value (unit: m).

**Figure 12:**
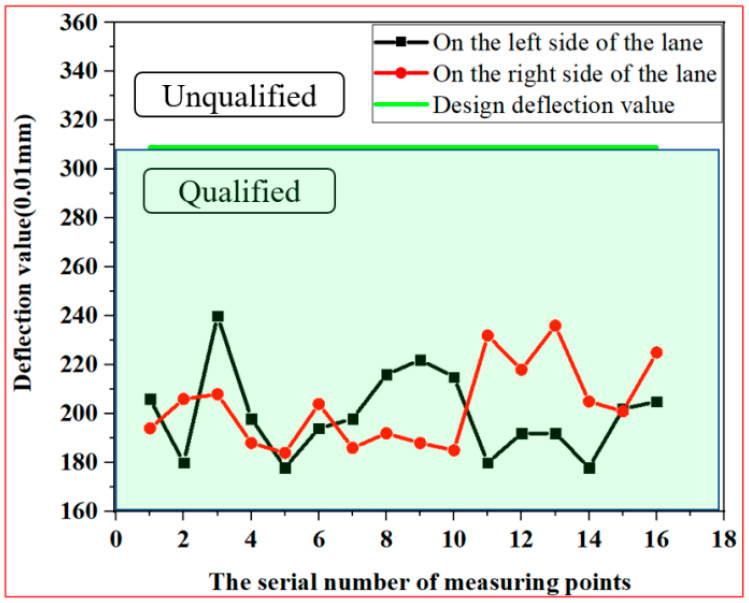
Bending settlement monitoring results.

A section of 300 m was selected for sampling, and the sampling was divided into two rows of points on the left and right. The points on the left were L1–L16, and the points on the right were R1–R16, with a total of 32 monitoring points, as shown in Figure 11. The deflection values of the test points are presented in Figure 12. The detection results were between 1.8 and 2.4 mm, all lower than the design value of 3.09 mm. Therefore, the design requirements were met.

## 5. Conclusions

In this study, LFCWM subgrade backfill material compaction, CBR, and shear tests were performed. The test results were carefully analyzed, and the following conclusions were drawn:

(1) Under different CAR conditions, the dry density first increased and then decreased with an increase in the water content. When CAR was 4:6, the optimum water content and maximum dry density reached the maximum values simultaneously, which were 10.1% and 2.03 g/cm^3^, respectively;
(2) With the increase in CAR, CBR increased first and then decreased, and when CAR was 4:6, CBR reached a maximum value of 42.5%;
(3) With the increase in shear displacement, the variation tendency of shear stress showed a rapid increase at first, then a slow decrease, and finally tended to be stable;
(4) With the increase in CAR, the maximum shear strength first increased and then decreased, and reached its maximum value when CAR was 4:6;
(5) The shear strength of the mixture increased with an increase in normal stress;
(6) As the amount of coarse aggregate increased, the shear strength of the mixture increased and then decreased. When P_4.75_ was 40%, the shear strength of the LFCWM was the maximum.

## 6. Discussion

In this study, the characteristics of mixed materials such as construction waste as subgrade backfill were studied from a macroscopic point of view, and the phenomenon was macroscopically analyzed. However, its mechanism and constitutive relationship are still unclear. In the future, the mechanism of action of LFCWM will be deeply studied from the microscopic perspective, and its constitutive model will be built to reveal the root cause of the occurrence of the macro law.

## Figures and Tables

**Table 1:** Parameters of fly ash.

Composition	Unit	Result
Moisture	%	0.04
Loss on ignition	%	1.13
Specific surface area	m^2^/kg	513
Specific gravity	g/cm^3^	2.42
Fe_2_O_3_	%	3.16
CaO	%	0.45
MgO	%	0.64
AL_2_O_3_	%	28.68
SiO_2_	%	59.04

**Table 2:** Lime composition.

Composition	Calcium Hydroxide (%)	Calcium Oxide (%)	Calcium Carbonate (%)	Magnesium Oxide (%)
Content	11.16	85.54	0.52	0.21

**Table 3:** Compaction test group design table.

Test Type	CAR	Moisture Content				
		6%	8%	10%	12%	14%
Compaction test	3:7	A	A	A	A	A
	4:6	A	A	A	A	A
	5:5	A	A	A	A	A
	8:2	A	A	A	A	A

**Table 4:** CBR test design.

Test Type	CAR	Optimum Moisture Content	Experimental Group
**CBR test**	3:7	10%	A B C
	4:6	12%	A B C
	5:5	12%	A B C
	8:2	12%	A B C

**Table 5:** Influence of CAR on shear strength.

Normal Pressure	CAR			
	3:7	4:6	5:5	8:2
100 kPa	A	A	A	A

**Table 6:** Test design of the influence of normal stress on shear strength.

CAR	Proportion of P_4.75_	Normal Pressure		
		100 kPa	200 kPa	300 kPa
4:6	40%	A	A	A

**Table 7:** Experimental design of particle diameter against shear strength.

CAR	Normal Pressure	Proportion of P_4.75_			
		20%	40%	60%	80%
4:6	100 kPa	A	A	A	A

## Data Availability

The data presented in this study are available on request from the corresponding author.

## Nomenclature

CBR value	The load bearing capacity is characterized by the ability of the material to resist the local load compression deformation, and the standard gravel of high quality is adopted as the standard, and the CBR value is expressed by their relative ratio
CAR	Cement-aggregate ratio
LFCWM	Lime-fly ash construction waste mixture
